# Criteria for identifying the molecular basis of the engram (CaMKII, PKMzeta)

**DOI:** 10.1186/s13041-017-0337-4

**Published:** 2017-11-29

**Authors:** John Lisman

**Affiliations:** Department of Biology, Brandeis University, Waltham, MA 02453 USA

**Keywords:** Herpes simplex virus, K42 M, Place aversion, Occlusion, Memory

## Abstract

The engram refers to the molecular changes by which a memory is stored in the brain. Substantial evidence suggests that memory involves learning-dependent changes at synapses, a process termed long-term potentiation (LTP). Thus, understanding the storages process that underlies LTP may provide insight into how the engram is stored. LTP involves induction, maintenance (storage), and expression sub-processes; special tests are required to specifically reveal properties of the storage process. The strongest of these is the Erasure test in which a transiently applied agent that attacks a putative storage molecule may lead to persistent erasure of previously induced LTP/memory. Two major hypotheses have been proposed for LTP/memory storage: the CaMKII and PKM-zeta hypotheses. After discussing the tests that can be used to identify the engram (Necessity test, Saturation/Occlusion test, Erasure test), the status of these hypotheses is evaluated, based on the literature on LTP and memory-guided behavior. Review of the literature indicates that all three tests noted above support the CaMKII hypothesis when done at both the LTP level and at the behavioral level. Taken together, the results strongly suggest that the engram is stored by an LTP process in which CaMKII is a critical memory storage molecule.

## Introduction

During learning, our brains are modified in such a way that the learned information can later be recalled, even many years later. The molecular modifications that store that information form the engram. Those modifications are likely to be contained in only a subset of neurons, and recent experiments confirm this directly. The experiments that identify the neurons that store the engram take advantage of the fact that iμμediate early genes are turned on in the subset of neurons that are strongly activated during learning. By linking expression of channel-rhodopsin to these genes, it has become possible to visualize and manipulate the activity of this subset. The key experimental result is that optogenetically exciting these cells elicits the behavior expected of memory recall [1]. It can therefore be concluded that the optogenetically excited cells either contain the engram or excite cells that do.

For the engram to mediate the recall process, the storage processes of the engram must affect neuronal signaling by an “expression process.” This might occur by making the cells that contain the engram more excitable (e.g., by modifying intrinsic non-synaptic conductances); alternatively, it may occur through modifications of synaptic function. Since the discovery of long-term potentiation (LTP), an activity-dependent and long-lasting increase of synaptic strength, it has been suspected that the engram involves changes in synaptic signaling mediated by an LTP-like process [2, 3]. The connection between LTP and memory is now supported by multiple lines of evidence [4–6] (but see [7]). Furthermore, LTP has been found to have properties that make it very well suited as a memory mechanism. First, analysis of LTP has shown that it enables the storage of vast amounts of information. Each of the over 10,000 synapses on a cell can be modified by LTP in a synapse-specific manner [8]. Gradations in synaptic strength vary over a 10-fold range (~ 3 bits of information) [9]. Therefore, if one considers just the CA3 region of the hippocampus, a region strongly implicated in episodic memory, the 3 million CA3 pyramidal cells in humans [10] contain about 30 billion synapses, thus making the storage of 100 billion bits of information possible. Second, LTP has been demonstrated [11] to have the Hebbian properties required to form meaningful associations in neuronal networks (LTP occurs at a synapse if there is both presynaptic activity and strong postsynaptic depolarization). Thus LTP has the desired properties to encode memory.

It follows that, in order to understand the molecular basis of the engram, it is important to identify the molecular processes responsible for the information storage that underlies the maintenance of LTP. When we consider how genetic memory is stored, the answer is rather simple: most genetic information is stored in the base sequences of DNA. It is natural to wonder whether the mechanisms responsible for storing the engram will be similarly simple. Because the criteria for identifying the biochemical basis of the engram have not been previously articulated, I will start by discussing appropriate criteria. I will then use these criteria to evaluate two major hypotheses for engram storage: the CaMKII (Calcium-Calmodulin Protein Kinase type II) hypothesis [12, 13] and the PKM-zeta (Protein Kinase M – zeta) hypothesis [14, 15]. Other hypotheses [16, 17] that have not received as much investigation will be not be discussed.

### Induction, maintenance, and expression processes that underlie late LTP

After LTP is induced, a variety of presynaptic and postsynaptic changes can produce short-lasting changes in synaptic transmission. Some of these may last only for seconds, but even weak induction protocols produce potentiation that can last for many minutes. The potentiation evident during the first 30 min after induction is generally referred to as early LTP. If the induction conditions are sufficiently strong, early LTP is followed by biochemically and structurally different processes that produce stable strengthening of the synapse; these processes are referred to as late LTP. Notably, protein synthesis inhibitors block late LTP, but not early LTP [18].

**Fig. 1:**
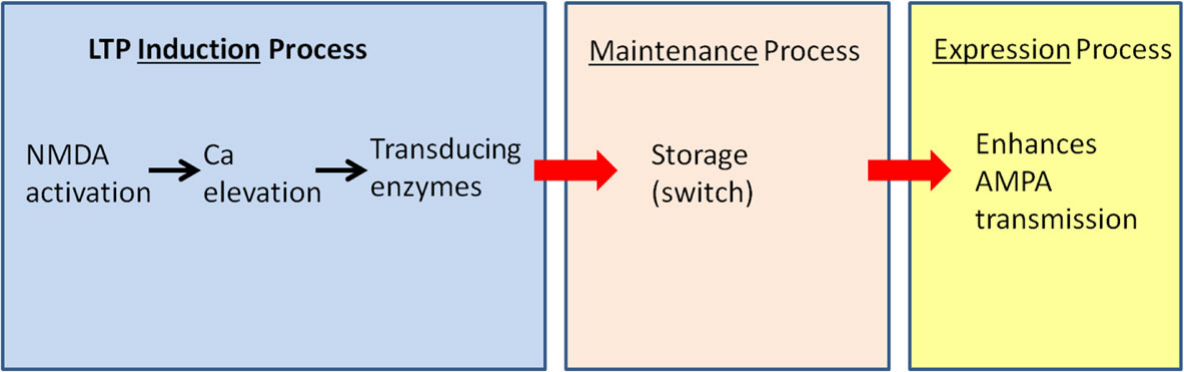
Schematic of the three subprocesses in LTP. The engram is stored by the maintenance process and is specific for each of ~ 10,000 synapses in a neuron

The processes that underlie the LTP can be classified into three functionally different categories: induction, maintenance, and expression processes (Fig. 1). Induction refers to events that occur near the time of stimulation and that trigger the downstream maintenance and expression processes. For example, because late LTP requires protein synthesis, the mechanisms that turn on this synthesis would be considered induction processes. The maintenance process is what underlies storage of the engram. Finally, via expression processes, the maintenance process leads to potentiation of the current through the AMPA (Alpha-amino-3-hydoxy-5-methyl-4-isoxazolepropionic acid) type of glutamate-activated ion channels, thereby leading to the observed potentiation of the EPSP (excitatory postsynaptic potentials). Expression processes could in principle be simple. For instance, if memory maintenance was due to the amount of activated kinase at the synapse, expression could simply be the phosphorylation of AMPA channels by the kinase. On the other hand, expression could be more complex and could involve a kinase-initiated cascade that leads to enhanced AMPA transmission through multiple steps. The cascade might work to enhance delivery of channels to the synapse and/or to enhance the number of structural slots capable of anchoring the channels at the synapse. Indeed, given the evidence that late LTP involves structural enlargement of the synapse [19, 20], it would seem that expression mechanisms that couple storage process to structural changes must be present.

In searching for the molecular basis of the engram, it is key to identify the mechanisms that specifically underlie the LTP maintenance process. That said, less-specific tests can also be useful; notably, if knocking out a protein reveals that it is not necessary for LTP, then it certainly cannot be part of the maintenance process. For this reason, the following section discusses three types of tests (Necessary, Saturation/Occlusion, and Erasure tests) used to explore the role of molecules in LTP and learning, even though only the Erasure test is powerful enough to specifically identify a role of a molecule in memory maintenance.

### Experimental tests that distinguish induction, maintenance, and expression processes

#### Necessary test

A commonly used test for determining whether a molecule is involved in LTP is to pharmacologically inhibit a molecule or to genetically knock it out. If this has no effect on LTP, then the molecule cannot be necessary for any LTP subprocess. If LTP is reduced or blocked, the molecule must have a role in one or more of the LTP subprocesses.

Some inhibitors have no effect on the earliest phases of LTP but block late LTP. It has been tempting to conclude that the targeted protein is therefore responsible for memory maintenance, but this is not a correct conclusion. The processes responsible for early and late LTP are biochemically very different, so finding an agent that selectively affects late LTP is not surprising given that the agent could be affecting events required for the *induction* of late LTP, but not early LTP (e.g., protein synthesis). Thus selective effects on late LTP do *not* imply a role of a target protein in the *maintenance* of late LTP. For this reason, the Necessary test can rule out the role of a protein in storing the engram but cannot provide positive evidence for such a role.

#### Saturation/occlusion test

In this test, an activated form of a protein is introduced into a neuron and the resulting change in the synaptic response is measured. If the protein enhances AMPAR-mediated transmission, it may or may not do so by the same biochemical process that occurs during LTP. This same/not-same issue can be investigated by studying the interaction of the two forms of potentiation. For instance, this can be done by producing potentiation with activated protein and then delivering a saturating LTP induction protocol (it is known that synaptic strength can be saturated). If the activated protein is indeed part of the normal LTP transduction pathway, subsequent delivery of an LTP induction protocol should have no effect (or at least a smaller effect than normal). Alternatively, subsequently normal LTP induction would indicate that the potentiation mechanism utilized by the protein was not the same potentiation mechanism that occurs during LTP. A putative engram molecule must pass this test. However, any molecule necessary for the induction, maintenance, or expression process can pass this test. Thus this test does not provide specific information about the storage mechanism. Nevertheless, the test is useful because it can rule out proteins that potentiate transmission by a process different from that which occurs during LTP/memory.

#### Erasure test

This is the sole test that is powerful enough to prove that a molecule is involved in storage of the engram. In this test, LTP is induced. Later, some kind of pharmacological or genetically expressed agent is used to attack the putative memory molecule. One then determines whether this reduces LTP. Since the agent was applied after LTP induction, any observed reduction cannot be due to an effect on induction processes. This reduction must be due to either an effect on maintenance or expression processes; removal of the agent can determine which is the case. If an expression process was affected, the remaining maintenance process will restore LTP. On the other hand, if the engram itself was destroyed, LTP will not recover (i.e., erasure occurred). One caveat, however, remains: a persistent reduction in LTP might be due to damage to the cell rather than erasure. It is thus critical to rule this out by showing that LTP can be *reinduced*. If this can be accomplished, it rules out damage of learning and recall processes and indicates that the memory maintenance process was indeed erased, not simply damaged. If a hypothesis passes this form of the Erasure test, it is appropriate to conclude that engram “erasure” occurred and that the protein being targeted is a required molecular component of the engram.

Unfortunately, proper conduct of the erasure test poses a technical difficulty. As noted above, it is crucial that the agent used to attack the putative memory molecule be applied and then *removed* before further testing. If the agent is not removed, a decrease in LTP or memory could be attributed to effects on the expression process rather than the maintenance process. Thus, identifying the molecular basis of the engram requires a method that allows an agent to be both applied and then removed before subsequent testing, a requirement not easily met when molecularly specific genetic methods are utilized.

### Using the above tests to evaluate the PKM-zeta model

PKM-zeta is a constitutively active type of atypical protein kinase C. It is synthesized for long periods after LTP induction [21]. It became a particularly promising engram candidate because a peptide inhibitor of this kinase, ZIP, produced powerful interference with LTP and memory maintenance in a variety of systems [22].

#### Necessary test

The concentration of ZIP used in in vivo experiments was several orders of magnitude greater than needed in slice experiments, raising questions about specificity [23]. Thus, confirmation of the PKM-zeta hypothesis with more specific genetic tools has been desirable. The first genetic experiments showed that PKM-zeta failed the Necessary test: knockout of PKM-zeta had little effect on LTP or memory [24, 25]. Moreover, the electrophysiological effects of ZIP were still seen in the knockout, indicating off-target effects. These results appeared to rule out a simple PKM-zeta hypothesis. Fortunately, more specific methods have now been brought to bear on the problem. Recent work has used genetically based antisense or dominant-negative approaches [15]. Using these methods, it was found that inhibition of PKM-zeta reduced late LTP and memory performance [15, 21, 26]. These molecular approaches are more specific than ZIP and leave little doubt that PKM-zeta has an important role in LTP. However, to account for all of the data, a more complex hypothesis is required. It has been suggested that, when PKM-zeta is knocked out, a related atypical Protein Kinase C (PKC-lambda) takes over [15]. Confirmation of this possibility awaits results with knockout of both kinases.

#### Occlusion test

**Fig. 2:**
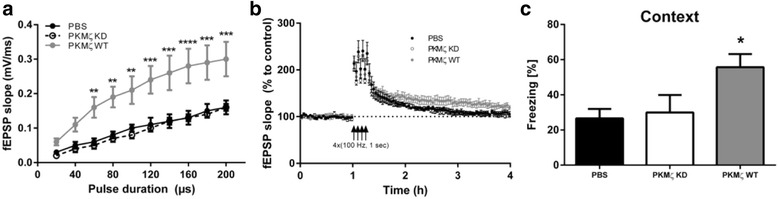
Occlusion test. AAV virus was used to overexpress PKM-zeta in vivo. **a** Overexpression of WT (wild type) kinase, but not the kinase dead (KD), form enhanced synaptic transmission. **b** Overexpression of WT kinase enhanced late LTP; i.e., occlusion did not occur. **c** Overexpression of WT enhanced contextual fear, measured 1 week after learning; i.e., occlusion did not occur. Data from [28]

Using a slice preparation, it was found that introduction of active PKM-zeta produces potentiation and that it then becomes impossible to produce LTP [27]. These results thus show the saturation/occlusion expected if PKM-zeta is important in LTP. However, quite different results have been obtained in two studies that used an in vivo approach and genetic overexpression of the enzyme. It was found that synaptic strength was increased (Fig. 2a) as expected but that LTP and memory were also *increased*, contrary to the prediction of occlusion (Fig. 2b, c) [28] (for similar effects see [26]). On the basis of this failure of occlusion, it was concluded that PKM-zeta is not critical for maintenance but is instead a modulator of LTP [28].

#### Erasure test

In an elegant application of the erasure test, it was shown that application of ZIP to the hippocampus in vivo could erase conditioned place avoidance [22]. This erasure persisted long after ZIP injection; it is therefore unlikely that erasure was due to the continued presence of ZIP. It thus appeared that PKM-zeta had passed the critical erasure test. However, recent work has identified major problems with the specificity of ZIP. When activity of endogenous PKM-zeta was measured in live cells, it was found that ZIP was an ineffective inhibitor [29]. Other work showed that ZIP can have toxic effects [30] and has powerful effects on processes other than synaptic transmission [31]. Taken together, these results make it difficult to use ZIP to determine the molecular basis of the engram.

**Fig. 3:**
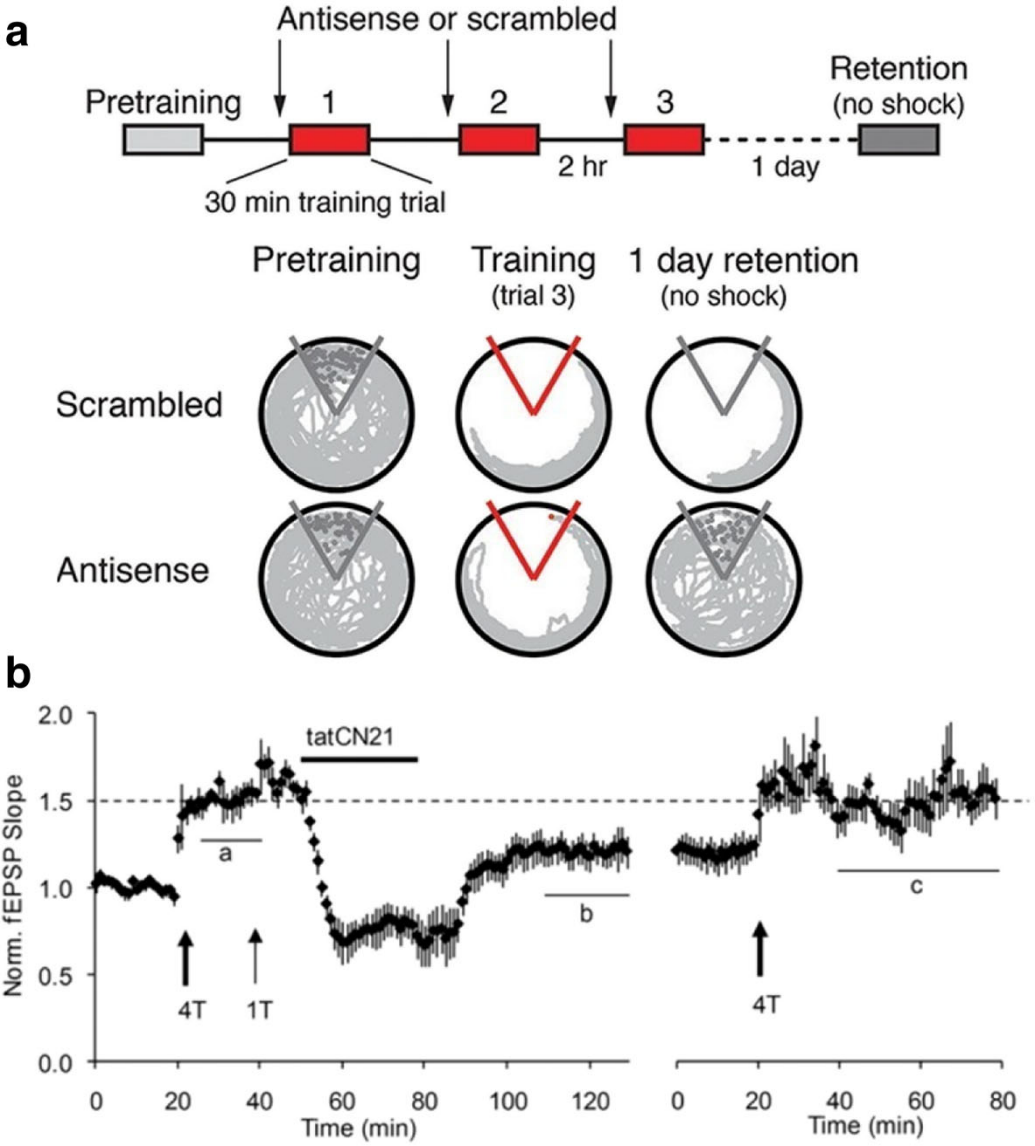
Use of the erasure test. **a** (*Top*) Protocol for testing the effect of PKM-zeta antisense (injected into hippocampus) on 1-day memory. (*Bottom*) *Grey lines* show track of rat on rotating platform that moved the rat into triangular shock zone defined relative to the room. After injection of scrambled DNA, the rat learned to avoid the shock zone and remembered 1 day later. If antisense was injected into the brain during multiple phases of the learning process, 1-day retention was abolished. Because the antisense was present during learning and probably also during retention, the failure of memory may be due to effects on induction or expression processes and thus do not provide specific information about the maintenance process. From [15] **b.** (*left*) Maximal LTP was induced by 4 tetani delivered to the CA1 region of a hippocampal slice. Bath application of tatCN21, a peptide that interferes with CaMKII function, produced a decrease in response that persisted after removal of tatCN21. Erasure of LTP was confirmed by the fact that LTP could then be reinduced (*right*). From [50]

New methods have been used to study PKM-zeta’s role in LTP/memory based on improved pharmacological agents and genetic methods [15, 21]. However, none of these studies have yet conducted the Erasure test as outlined above (removal on the attacking substance), a requirement that was met in the study that originally provided strong support for the PKM-zeta hypothesis [22]. Notably, in a recent study, application of anti-sense PKM-zeta to the brain reduced the learning-dependent increase in PKM-zeta and reduced memory performance [21]. However, in the experiments of Fig. 3a, the antisense was present during learning and was probably still present during the testing of 1-day memory (virally expressed proteins are likely to persist for at least a day). Thus, these agents may well have affected induction and/or expression processes. This problem with the design of the Erasure test indicates that no firm conclusion can yet be reached about the role of PKM-zeta in the maintenance process.

### Using the above tests to evaluate the CaMKII model

CaMKII is one of the most abundant brain proteins. It exists in the cytoplasm at high concentration but is further concentrated in the postsynaptic density of glutamatergic synapses where it is a major protein [32]. The kinase holoenzyme consists of two rings of six subunits, each of which is catalytic. When Ca^2+^ enters the synapse during LTP induction, it leads to efficient activation of the CaMKII within spines (reviewed in [33]). This activation produces autophosphorylation of T286 sites on the kinase, a phoshorylation that makes the kinase persistently active even after Ca^2+^ levels fall [34]. Most of the 1000 CaMKII molecules [35] within a spine are inactivated within minutes [36], but a small pool (on the order of 50) can bind to the PSD and persist there for at least an hour [37]. Recent work indicates that the PSD itself has two compartments, a core region directly juxtaposed to the postsynaptic membrane and the more distant pallium region [38]. Most of the 50 or more CaMKII molecules in the PSD are in the pallium, but a few (on the order of 10) are in the core [39], where they may be bound to NMDARs [40, 41]. There are reasons to think that it is this pool that is most important in LTP [42]. For a review of CaMKII function in LTP, see [33].

#### Necessary test

Knockout of CaMKII-alpha [43] or knockin of a mutant form that cannot autophosphorylate (T286A) [44] or is catalytically dead (K42 M) [45] greatly reduces LTP and memory. These results indicate that CaMKII must have a critical role in induction, maintenance, or expression processes. Studies of knockout and knockin mutations showed that the animals had strong deficits in memory-guided behavior, consistent with a critical role of LTP in memory.

#### Occlusion/saturation test

Intracellular application of the catalytic region of CaMKII potentiates transmission and strongly inhibits the induction of subsequent LTP [46]. Similar results were obtained by overexpression of activated CaMKII holoenzyme (T286D/T305A/T306A) [47]. These results thus suggest that activated CaMKII has powerful capability to potentiate AMPAR transmission and that this capability is utilized during the maintenance phase of LTP.

The occlusion/saturation test has been utilized to study behaviorally -defined memory and, specifically, the role of LTP in memory. In a critical set of experiments [4], learning occurred and was later followed by saturating induction of LTP in the dentate gyrus. This produced a strong deficit in subsequent memory behavior, as expected if saturation degraded memory. Stated differently, if memory depends on the differential strength of synapses, strengthening them all would be expected to degrade memory.

**Fig. 4:**
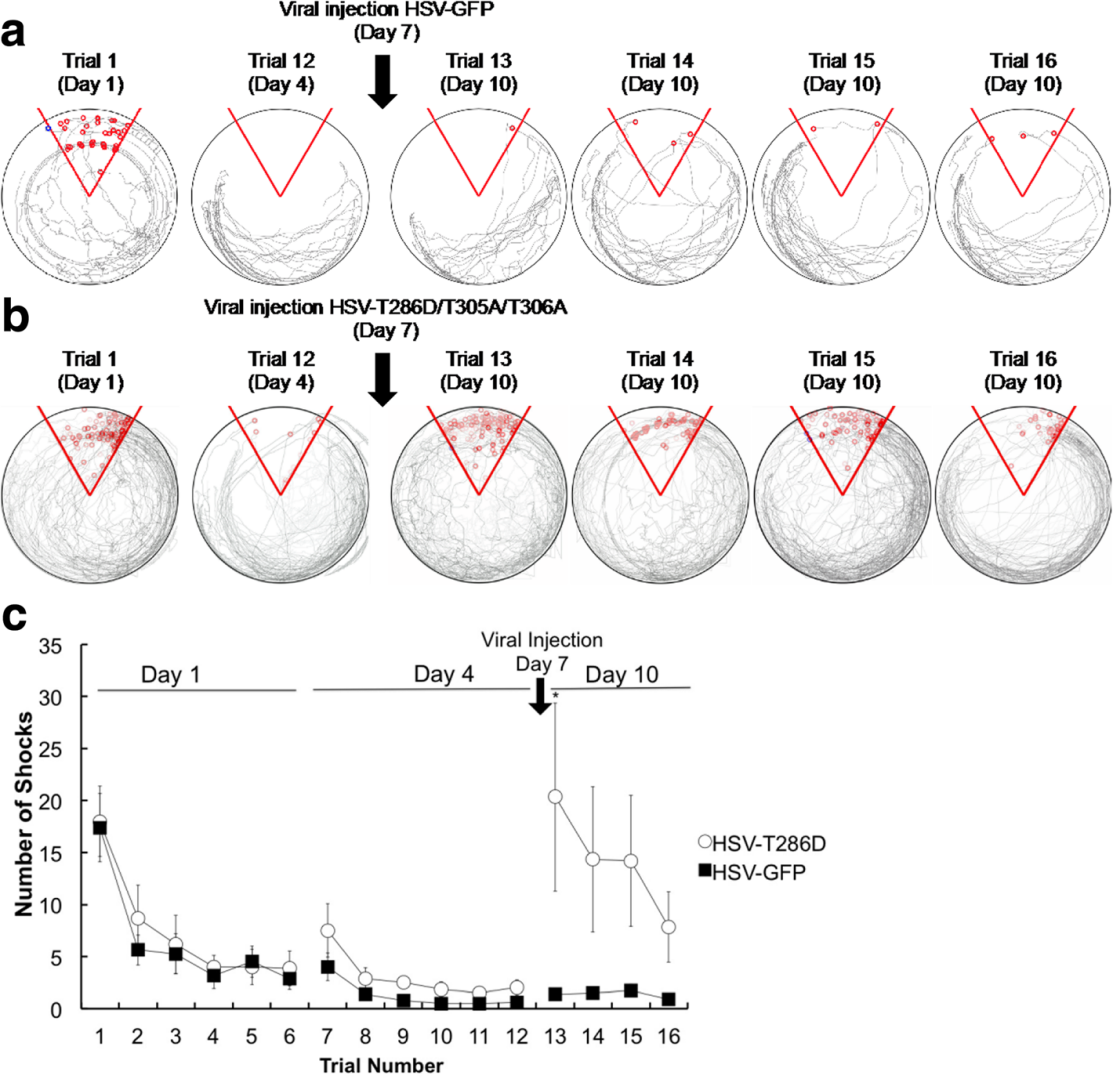
Saturation test. **a** Path of rats (*grey*; superposition of six experiments) on circular platform before and after injection of control virus expressing GFP; shock zone (*red pie shape*) and individual shocks given (small red circles). Trial 1 is first training trial on day 1; trial 12 is last training trial (on day 4); reduction in number of shocks indicates learning. Trial 13 tests memory retention on day 10, 3 days after viral injection. Trials 14–16 show a deficit in relearning on day 10. **b** Path of rats before and after injection of virus expressing activated CaMKII (T286D/T305A/T306A). **c** Summary data. After viral expression of T286D/T305A/T306A, memory was poor (*p* = 0.02, D = 0.75) (*n* = 6) compared to GFP controls. After viral expression of GFP, memory was strong in one group measured at day 10 (*n* = 2) and in another group measured at day 16 (*n* = 6) (the groups are not significantly different, so data is combined here as “day 10” (*n* = 8)). For CaMKII*-injected animals, memory on day 10 was not significantly different than memory on day 16 for K42 M-injected animals (*p* = 0.8096, D = 0.3333). A two-sample K-S test was used to determine statistical significance. Error bars represent mean ± SEM. Asterisks indicate statistical significance (*p* < 0.05; Kolmogorov–Smirnov test). Reprinted with permission from [48]

Recent work has used a conceptually related strategy to test the role of CaMKII in memory-guided behavior [48]. In these experiments, animals first learned a conditioned place avoidance task. Several days later, a Herpes Simplex viral vector (HSV) was used to deliver activated CaMKII (T286D/T305A/T306A) to the hippocampus. Prior work had showed that this mutant strongly potentiates synapses, driving them to saturation, as indicated by the inability to induce further potentiation using strong synaptic stimulation [47]. When memory was tested at the time of strong expression of activated CaMKII (3 days after viral injection), memory behavior was strongly inhibited (Fig. 4). These results thus support the concept that memory is mediated by an LTP-like process dependent on CaMKII.

#### Erasure test

The erasure test for LTP was conducted by bath application of a peptide (TatCN21) that inhibits CaMKII and interferes with its binding to the NMDAR [49]. Transient application of the peptide after LTP induction reversed established LTP, which could then be reinduced by an LTP induction protocol (Fig. 3b) [50].

These results suggest that LTP erasure occurred, but several lines of other experiments strengthen this conclusion. First, the ability of 20 μM of TatCN21 to interfere with the CaMKII/NMDAR complex in slices was confirmed biochemically [50]. Second, erasure produced by tatCN27, another CaMKIIN-derived peptide, was not simply due to LTD processes [51]. Third, the effect did not occur in neonatal animals that lack CaMKII-alpha in their PSDs [51]. Fourth, the erasure of LTP by tatCN21 was replicated [52] and demonstrated to be a postsynaptic effect. Fifth, it was shown that effects of tatCN21 were reduced in mice having a GluN2B mutation that interfered with the ability of CaMKII to bind to NMDARs [52]. These additional experiments, together with the primary results of Fig. 3b, make a strong case that CaMKII mediates the LTP storage process. For a full discussion of why other CaMKII inhibitors do not produce this effect seen in Fig. 3b, see [48] (briefly, only CN inhibitors at high concentration can interfere with the binding of CaMKII to GluN2B, NR2B NMDA receptor).

**Fig. 5:**
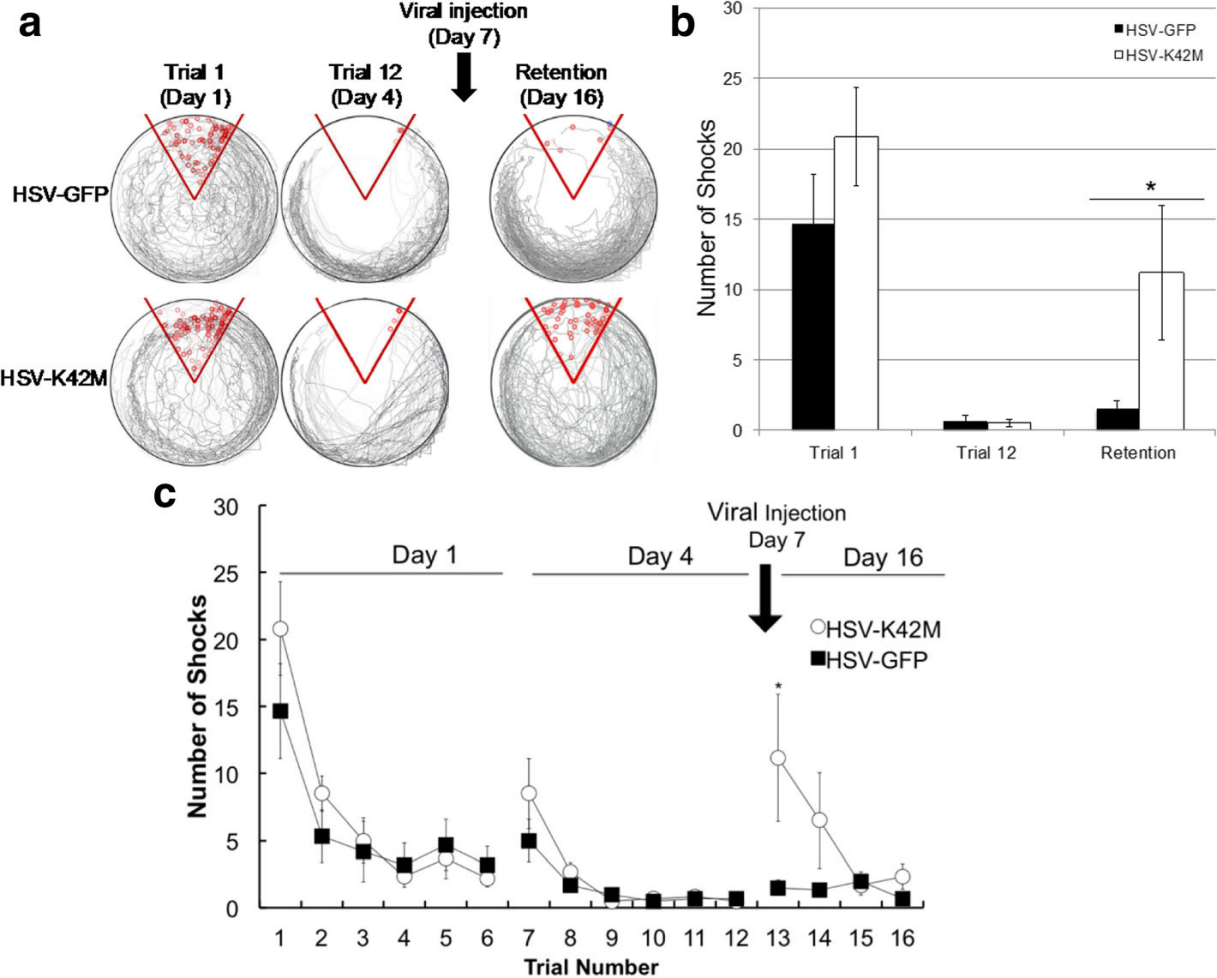
Erasure test. Memory was tested 9 days after virus injection (day 16), a time at which virally mediated protein expression had ended (Fig. 1b). **a** Superposition of paths of six rats (*top*). Memory is largely preserved after GFP expression but was largely erased (*bottom*) after expression of dominant-negative CaMKII (K42 M). **b,c** Summary data. A two-sample K-S test was used to determine statistical significance (*p* = 0.012, D = 0.83; n = 6). The differences on trials 1 and 7 (pre viral injection) between K42 M and GFP were not statistically significant (*p* = 0.81 and D = 0.33 for trial 1; *p* = 0.32 and D = 0.5 for trial 7). Error bars represent mean ± SEM. Asterisks indicate statistical significance (p < 0.05; Kolmogorov–Smirnov test). Reproduced with permission from [48]

In the most critical test of the CaMKII in memory, the Erasure test was used to determine whether interfering with CaMKII could erase a behaviorally defined memory. In these experiments, a dominant-negative form of CaMKII was expressed several days after learning. A requirement of the Erasure test is that the dominant negative be only transiently expressed. Such transient expression is a well-established property of HSV [53], a virus chosen for this reason. Memory was tested 10 days after viral transfection, a time at which CaMKII expression was demonstrated to have ceased. As shown in Fig. 5, memory was strongly reduced. Given that the dominant negative was no longer present, this effect is unlikely to involve expression processes. However, the reduction of memory might have resulted from damage to the hippocampus, but the fact that relearning could occur argues strongly against this. In an important further control, the same viral strategy was used to express wild-type CaMKII. In this case (the difference being only one amino acid), no erasure was produced. These results thus suggest that memory, like LTP, can be erased by interference with CaMKII function.

### Additional criteria

#### Mechanisms of stability

**Fig. 6:**
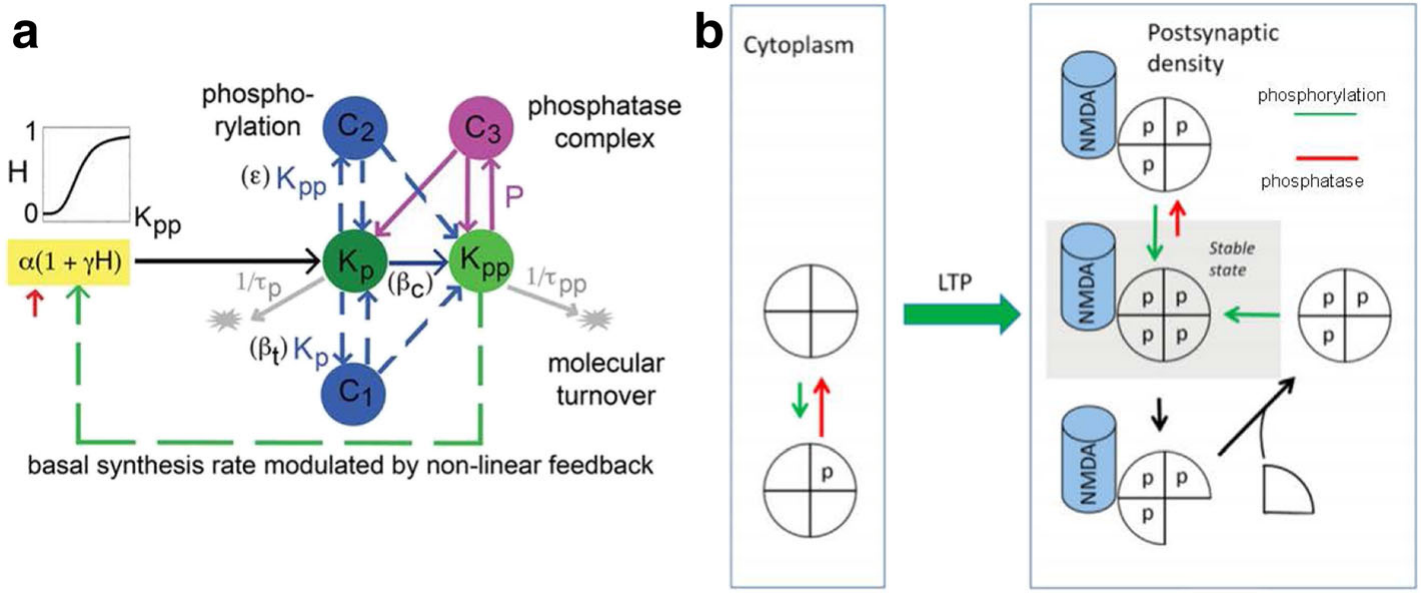
Models of stable information storage by a molecular switch. **a** PKM-zeta model. Black arrow is protein synthesis of PKM-zeta that occurs during late phase of LTP. It is postulated that singly phosphorylated kinase (Kp) can be autophosphorylated to produce doubly phosphorylated kinase (Kpp), which then stimulates further synthesis of PKM-zeta and stable information storage. How synapse specificity is achieved is not specified. The possibility that atypical PKCs undergo such regulated phosphorylation has not been confirmed in recent work [62]. From [55]. **b** CaMKII model. LTP induction leads to autophosphorylation of CaMKII T286, which leads to persistent activation of the kinase and binding to the NMDA channel within the potentiated spine, thereby establishing synapse-specificity. If a subunit gets dephosphorylated (*upward red arrow*), the subunit is rephosphorylated by a neighboring active subunit. Protein turnover (*downward black arrow*) occurs by subunit exchange. A newly inserted unphosphorylated subunit will be phosphorylated by a neighboring subunit. Thus the switch will be stable despite phosphatase activity and protein turnover. From [60]

A satisfactory molecular theory of memory storage needs to address the question of how the stability of memory is achieved. Memory lasts a long time compared to the lifetime of synaptic proteins, all of which undergo turnover within a week or less [54]. Thus, particular mechanisms must exist to ensure stable information storage by unstable molecules. Solutions to this problem have been proposed for both the PKM-zeta [55] and CaMKII models [12, 56] (Fig. 6; for explanations, see caption). At the core of both models is the concept of a positive feedback chemical system that can sustain the on-state of a switch. Because the system contains multiple molecules (subunits in the case of CaMKII) and because switch function depends on a multi-molecular system, individual molecules can be replaced by protein turnover without loss of information.

#### Persistence of molecular modification

LTP induction leads to persistent translocation of CaMKII to the PSD (measured 1 h after induction) [37] and to phosphorylation that can last for at least many hours [57]. It has not yet been possible to follow the state of CaMKII on the longer time scale. However, basal conditions measured in hippocampal slices may reflect LTP processes that occurred days before while the animal was still alive. On the simplest model, synapses start as silent (no AMPAR conductance) and LTP-like processes lead to enhancement of the AMPAR conductance. Consistent with such a model, the complex of CaMKII with the NMDAR is found under basal conditions in slices and reduction in this complex is associated with reduction in AMPAR conductance [50]. Importantly, in the presence of mutations that block CaMKII interaction with NMDAR, there is no basal AMPAR-mediated transmission [58]. A major advance would be the development of FRET methods that would allow the CaMKII bound to the NMDAR to be monitored in single spines over long periods.

In the case of PKM-zeta, recent work has monitored its learning-induced elevation over very long periods. Impressively, a 20% increase in total PKM-zeta in CA1 can be measured as long as 1 month after learning [21], making it the most persistent learning-produced biochemical change yet observed.

#### Synapse specificity

Given the evidence that LTP is synapse-specific (e.g., can occur at the active spine, but not at spines only a few microns away), a successful model should account for how such a high degree of localization can be achieved. In the case of CaMKII, a FRET-based reporter of activated CaMKII shows that this species is largely restricted to the stimulated spine, thus providing a local biochemical signal that can account for synapse specificity [36]. The local changes may include binding to NMDARs in that spine [41, 59], thereby forming a synapse-specific molecular engram within the postsynaptic density of the activated spine. This complex may then serve as a structural seed for the addition of other proteins, leading to trans-synaptic growth of synapse and the associated addition of AMPA channels [60].

Recent work on PKM-zeta demonstrates its role in nuclear signaling [61]. After LTP induction, PKM-zeta moves through the dendrite to the nucleus. It is active there in phosphorylating CREB binding protein (CBP). This, it is argued, might produce epigenetic changes necessary for long-term memory. However, the fact that active kinase is spreading through the dendrites to the nucleus poses a problem for any memory storage model because the active kinase could easily destroy the specific-specific action required for proper memory function. On the other hand, the spread of potentiation could contribute to a synaptic scaling function that is not synapse-specific. Indeed, such a role would help to account for the results of the occlusion test, which are more consistent with a role in scaling than synapse-specific memory storage.

## Conclusions

The molecular basis of memory storage is one of the most fundamental questions in cellular neuroscience. It is remarkable that such a fundamental question has remained unanswered. One reason for limited progress is the difficulty of conducting the key erasure test. This test requires not only target specificity, a specificity that is difficult to achieve in vivo by traditional pharmacological methods, but also temporal control: as noted above, proper execution of the erasure test requires that the agent used to attack a putative memory molecule must be introduced and then removed. These requirements for specificity and temporal control have now been met using the HSV system for viral delivery of dominant-negative CaMKII. The results clearly demonstrate memory erasure. Similar erasure had been previously achieved in slice experiments on LTP. Thus, a reasonable conclusion is that memory is stored by an LTP-like process that depends on CaMKII.

## Abbreviations

AMPA: α-amino-3-hydroxy-5-methyl-4-isoxazolepropionic acid (receptor)
CaMKII: Calcium-Calmodulin-dependent Protein Kinase Type II
EPSP: Excitatory postsynaptic potential
HSV: Herpes simplex virus
KD: Kinase dead
Kp: Kinase phosphorylated
Kpp: Kinase doubly phosphorylated
LTP: Long-term potentiation
NMDA: N-methy-D-aspartate (receptor)
PKC: Protein kinase C
PKM: Protein kinase M
PSD: Postsynaptic density
WT: Wild type
